# Advances and Challenges of Liposome Assisted Drug Delivery

**DOI:** 10.3389/fphar.2015.00286

**Published:** 2015-12-01

**Authors:** Lisa Sercombe, Tejaswi Veerati, Fatemeh Moheimani, Sherry Y. Wu, Anil K. Sood, Susan Hua

**Affiliations:** ^1^The School of Biomedical Sciences and Pharmacy, The University of NewcastleCallaghan, NSW, Australia; ^2^Hunter Medical Research Institute, New Lambton HeightsNSW, Australia; ^3^Department of Gynecologic Oncology, The University of Texas MD Anderson Cancer CenterHouston, TX, USA; ^4^Department of Biochemistry and Cell Biology, Rice UniversityHouston, TX, USA; ^5^Center for RNA Interference and Non-Coding RNAs, The University of Texas MD Anderson Cancer CenterHouston, TX, USA; ^6^Department of Cancer Biology, The University of Texas MD Anderson Cancer CenterHouston, TX, USA

**Keywords:** liposomes, drug delivery, lipid-based drug delivery system, nanotechnology, biological challenges, translation, accelerated blood clearance, complement activation–related pseudoallergy

## Abstract

The application of liposomes to assist drug delivery has already had a major impact on many biomedical areas. They have been shown to be beneficial for stabilizing therapeutic compounds, overcoming obstacles to cellular and tissue uptake, and improving biodistribution of compounds to target sites *in vivo*. This enables effective delivery of encapsulated compounds to target sites while minimizing systemic toxicity. Liposomes present as an attractive delivery system due to their flexible physicochemical and biophysical properties, which allow easy manipulation to address different delivery considerations. Despite considerable research in the last 50 years and the plethora of positive results in preclinical studies, the clinical translation of liposome assisted drug delivery platforms has progressed incrementally. In this review, we will discuss the advances in liposome assisted drug delivery, biological challenges that still remain, and current clinical and experimental use of liposomes for biomedical applications. The translational obstacles of liposomal technology will also be presented.

## Introduction

**Figure 1:**
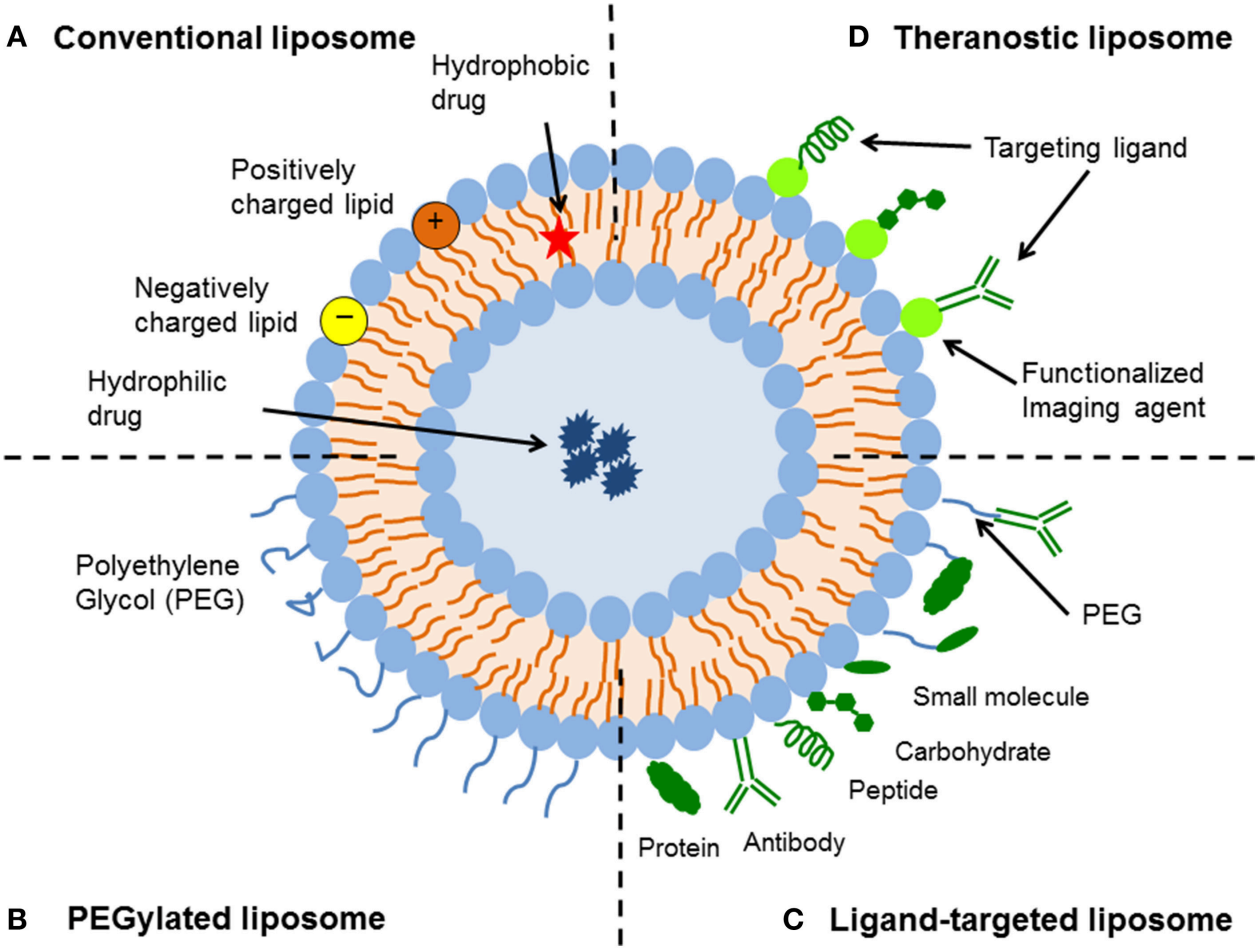
**Schematic representation of the different types of liposomal drug delivery systems. (A)** Conventional liposome—Liposomes consist of a lipid bilayer that can be composed of cationic, anionic, or neutral (phospho)lipids and cholesterol, which encloses an aqueous core. Both the lipid bilayer and the aqueous space can incorporate hydrophobic or hydrophilic compounds, respectively. **(B)** PEGylated liposome—Liposome characteristics and behavior *in vivo* can be modified by addition of a hydrophilic polymer coating, polyethylene glycol (PEG), to the liposome surface to confer steric stabilization. **(C)** Ligand-targeted liposome—Liposomes can be used for specific targeting by attaching ligands (e.g., antibodies, peptides, and carbohydrates) to its surface or to the terminal end of the attached PEG chains. **(D)** Theranostic liposome—A single system consist of a nanoparticle, a targeting element, an imaging component, and a therapeutic component.

Liposomes are the most common and well-investigated nanocarriers for targeted drug delivery. They have improved therapies for a range of biomedical applications by stabilizing therapeutic compounds, overcoming obstacles to cellular and tissue uptake, and improving biodistribution of compounds to target sites *in vivo* (Koning and Storm, 2003; Metselaar and Storm, 2005; Ding et al., 2006; Hua and Wu, 2013). Liposomes are defined as phospholipid vesicles consisting of one or more concentric lipid bilayers enclosing discrete aqueous spaces. The unique ability of liposomal systems to entrap both lipophilic and hydrophilic compounds enables a diverse range of drugs to be encapsulated by these vesicles. Hydrophobic molecules are inserted into the bilayer membrane, and hydrophilic molecules can be entrapped in the aqueous center (Koning and Storm, 2003; Metselaar and Storm, 2005; Ding et al., 2006; Hua and Wu, 2013; Figure 1). Furthermore, the large aqueous center and biocompatible lipid exterior permits the delivery of a variety of macromolecules, such as DNA, proteins and imaging agents (Ulrich, 2002; Monteiro et al., 2014). As a drug delivery system, liposomes offer several advantages including biocompatibility, capacity for self-assembly, ability to carry large drug payloads, and a wide range of physicochemical and biophysical properties that can be modified to control their biological characteristics (Koning and Storm, 2003; Metselaar and Storm, 2005; Ding et al., 2006; Hua and Wu, 2013). Liposomal formulations are characterized by properties such as particle size, charge, number of lamellae, lipid composition, and surface modification with polymers and ligands—these all govern their stability *in vitro* and *in vivo* (Hua and Wu, 2013; Monteiro et al., 2014). Encapsulation within liposomes protects compounds from early inactivation, degradation and dilution in the circulation (Ulrich, 2002). Liposomes are generally considered to be pharmacologically inactive with minimal toxicity, as they tend to be composed of natural phospholipids (Koning and Storm, 2003; Metselaar and Storm, 2005; Ding et al., 2006; Hua and Wu, 2013); however increasing number of studies have shown that liposomes are not as immunologically inert as once suggested (Szebeni and Moghimi, 2009). Despite the success of liposomal formulations *in vivo*, their translation into the clinic has progressed incrementally. This review will address the advances, biological challenges, biomedical applications, and translational obstacles of liposomal technology.

## Types of liposomal drug delivery platforms

In general, there are four key types of liposomal delivery systems—conventional liposomes, sterically-stabilized liposomes, ligand-targeted liposomes, and a combination of the above (Figure 1). Conventional liposomes were the first generation of liposomes to be developed. They consist of a lipid bilayer that can be composed of cationic, anionic, or neutral (phospho)lipids and cholesterol, which encloses an aqueous volume (Figure 1). Research on the clinical potential of conventional liposomes began in the 1980s, whereby liposomal delivery proved useful for improving the therapeutic index of encapsulated drugs, such as doxorubicin and amphotericin (Gabizon et al., 1982; Koning and Storm, 2003; Metselaar and Storm, 2005; Ding et al., 2006; Hua and Wu, 2013). Conventional liposomal formulations reduced the toxicity of compounds *in vivo*, through modifying pharmacokinetics and biodistribution to enhance drug delivery to diseased tissue in comparison to free drug. However, the delivery system was prone to rapid elimination from the bloodstream, therefore limiting its therapeutic efficacy (Gabizon et al., 1991, 1994). This rapid clearance was due to opsonization of plasma components and uptake by fixed macrophages of the reticuloendothelial system (RES), mainly in the liver and spleen (Hua and Wu, 2013).

To improve liposome stability and enhance their circulation times in the blood, sterically-stabilized liposomes were introduced. The hydrophilic polymer, polyethylene glycol (PEG), has been shown to be the optimal choice for obtaining sterically-stabilized liposomes (Figure 1). The establishment of a steric barrier improves the efficacy of encapsulated agents by reducing *in vivo* opsonization with serum components, and the rapid recognition and uptake by the RES. This not only reduces the elimination of drugs by prolonging blood circulation and providing accumulation at pathological sites, but also attenuates side effects (Torchilin et al., 1992; Northfelt et al., 1996; Ishida et al., 2001b). Steric stabilization strongly influences the pharmacokinetics of liposomes (Gabizon et al., 1993), with reported half-lives varying from 2 to 24 h in rodents (mice and rats) and as high as 45 h in humans, depending on the particle size and the characteristics of the coating polymer (Allen, 1994; Moghimi and Szebeni, 2003). While coating liposomes with PEG results in prolonged circulation times, there can be an offsetting reduction in the ability to interact with the intended targets (Willis and Forssen, 1998; Ulrich, 2002).

Ligand-targeted liposomes offer a vast potential for site-specific delivery of drugs to designated cell types or organs *in vivo*, which selectively express or over-express specific ligands (e.g., receptors or cell adhesion molecules) at the site of disease (Willis and Forssen, 1998; Hua, 2013). Many types of ligands are available, such as antibodies, peptides/proteins and carbohydrates (Figure 1). The coupling of antibodies, particularly monoclonal antibodies, to create immunoliposomes represents one of the more versatile ligands that can be affixed to liposome surfaces (Bendas, 2001; Puri et al., 2009). One of the advantages of using monoclonal antibodies is their stability and higher binding avidity because of the presence of two binding sites on the molecule. Since lipid assemblies are usually dynamic structures, surface-coupled ligands have a high motional freedom to position themselves for optimal substrate-interactions (Willis and Forssen, 1998). The limited *in vivo* performance of immunoliposomes, due to poor pharmacokinetics and immunogenicity, has been a major hurdle to achieving their potential as effective site-specific drug carriers (Puri et al., 2009). Therefore, newer generation of liposomes have utilized a combination of the above design platforms to further improve liposomal targeting and associated drug delivery (discussed in *Experimental use of liposomes for biomedical applications*). For example, integrating target-specific binding of immunoliposomes with the steric stabilization of PEG (thereby creating long-circulating immunoliposomes) has significantly improved the pharmacokinetics of immunoliposomes (Maruyama, 2002). Overall as a drug delivery platform, liposomes offer a dynamic and adaptable technology for enhancing the systemic efficacy of therapeutics in various diseases.

## Biological challenges facing liposomal drug delivery systems

As with any foreign particle that enters the body, liposomes encounter multiple defense systems aimed at recognition, neutralization, and elimination of invading substances. These defenses include the RES, opsonization, and immunogenicity (Willis and Forssen, 1998). While these obstacles must be circumvented for optimal liposome function, other factors such as the enhanced permeability and retention (EPR) effect can be exploited to enhance drug delivery (Sawant and Torchilin, 2012).

### The reticuloendothelial system (RES) and liposome clearance

The RES is the main site of liposome accumulation following their systemic administration (Poste et al., 1976; Senior, 1987). Primary organs associated with the RES include the liver, spleen, kidney, lungs, bone marrow, and lymph nodes (Senior, 1987). The liver exhibits the largest capacity for liposomal uptake followed by the spleen, which can accumulate liposomes up to 10-fold higher than other RES organs (Chrai et al., 2002). The ability of the RES to sequester liposomes from the circulation is attributed to fenestrations in their microvasculature. Pore diameters in these capillaries can range from 100 to 800 nm, which is large enough for the extravasation and subsequent removal of most drug-loaded liposomes (50–1000 nm in size) (Sapra and Allen, 2003). Liposomes are cleared in the RES by resident macrophages via direct interactions with the phagocytic cells (Chrai et al., 2002). Uptake of liposomes by the RES is typically secondary to vesicle opsonization—that is, the adsorption of plasma proteins such as immunoglobulin, fibronectin, lipoproteins, and/or complement proteins onto the phospholipid membrane (Ishida et al., 2001a; Chrai et al., 2002). However, *in vitro* studies have demonstrated that liposomal clearance via macrophages can also occur in the absence of plasma proteins (Chrai et al., 2002).

The cells of the RES are also part of the innate immune system, which has raised the question of whether macrophage saturation by liposomes leads to immunosuppression and increases the risk of infections. Excessive liposome deposition in macrophages may impair their phagocytic capacity or modulate other cellular functions; however there have been no reports to date of clinically significant immune suppression at therapeutic doses of non-cytotoxic liposomes (Szebeni and Barenholz, 2009; Szebeni and Moghimi, 2009). The situation is different with anti-cancer liposomes that contain cytotoxic drugs, which are capable of inducing macrophage destruction. Although clinically important blockade of macrophage function in humans have not yet been demonstrated, there have been indirect signs that suggest the possibility of some immune suppression (Szebeni and Barenholz, 2009; Szebeni and Moghimi, 2009). For example, administration of pegylated liposomal doxorubicin (PLD) (Doxil®) in mice showed a dose-dependent clearance saturation effect due to partial blockade of the RES in the liver. This effect was not present after administration of a similar free doxorubicin dose or phospholipid dose in drug-free liposomes (Gabizon et al., 2002). In addition, administration of Doxil® in mice was reported to interfere with the clearance of bacteria from the blood, which was suggested to be due to macrophage suppression (Storm et al., 1998; Szebeni and Barenholz, 2009).

Conjugation of PEG polymers to the liposomal membrane is a key strategy for improving circulation times and preventing removal by the RES through steric stabilization (Oku and Namba, 1994; Ishida et al., 2001a). PEGylation creates a local surface concentration of highly hydrated groups, which sterically inhibits both electrostatic and hydrophobic reactions with plasma proteins and/or cells and thereby reduces liposomal uptake by the RES (Ishida et al., 2001a). The use of PEG significantly minimizes, but does not completely circumvent, liposomal uptake by the RES—with pathways independent of opsonization also possible (Laverman et al., 2001; Sawant and Torchilin, 2012).

### Opsonins and vesicle destabilization

The degree of interaction between liposomal drug delivery systems and plasma proteins is important in determining overall nanocarrier biodistribution, efficacy, and toxicity (Hua and Wu, 2013). Plasma proteins have been shown to play a pivotal role in liposomal clearance by the RES via opsonization, as well as in vesicular destabilization (Cullis et al., 1998). Opsonization of liposomes by serum proteins depends on a variety of factors including size, surface charge and stability (Cullis et al., 1998; Ishida et al., 2001a). The extent of this interaction has been shown to decrease with liposome size from 800 to 200 nm in diameter, as small liposomes cannot support opsonic activity (Chrai et al., 2002). This profound effect of liposome size on complement recognition can also affect liver uptake (Chrai et al., 2002). Generally, large unmodified liposomes are eliminated more rapidly than small, neutral, or positively charged liposomes (Oku and Namba, 1994; Laverman et al., 1999; Ulrich, 2002). Nevertheless, the presence of high electrostatic charge can still promote the interaction of liposomes with biomolecules that can serve as opsonins (Laverman et al., 1999; Ishida et al., 2001a). Previous investigation has revealed that large, charged liposomes are cleared within minutes by the liver and less than an hour by the spleen (Senior, 1987; Chrai et al., 2002). The inclusion of cholesterol is an important factor for increasing liposome stability and minimizing phospholipid exchange (Willis and Forssen, 1998). Incorporation of cholesterol into the liposomal membrane abates lipid exchange with other circulating structures (e.g., red blood cells and lipoproteins) that can cause the depletion of high phase transition temperature lipids and their replacement with less physiologically stable components (Willis and Forssen, 1998; Laverman et al., 1999; Ulrich, 2002). Integrating cholesterol into small (approximately 100 nm), electrostatically neutral liposomes has been shown to prolong circulation time in the range of several hours (Geng et al., 2014).

### The enhanced permeability and retention (EPR) effect

Liposomes that have evaded both the RES and opsonization are subjected to the EPR effect (Sawant and Torchilin, 2012; Nehoff et al., 2014). The EPR effect refers to the increased permeability of the vasculature that supplies pathological tissues (e.g., tumors and conditions involving inflammation). At these sites, deregulations in angiogenesis and/or the increased expression and activation of vascular permeability factors predominates (Nehoff et al., 2014), which leads to fenestrations that can range from 300 to 4700 nm. This allows liposomes to extravasate and accumulate by passive targeting (Hashizume et al., 2000). For example, inflammation results in a dramatic change in blood vessel permeability as the capillary vasculature undergoes structural remodeling to allow leukocyte diapedesis into the peripheral tissue (Klimuk et al., 1999; Hua, 2013). The width of the tight junctional regions between endothelial cells *in vivo* has been reported to range from 12 to 20 nm (Antohe et al., 2004), however exposure to inflammatory mediators increases permeability of the microvasculature, with the formation of gaps of up to 1 μm (Antohe et al., 2004). Pore sizes ranging from 0.2 to 1.2 μm have been observed, though the size and number of pores are dependent upon the microenvironment of the pathological site (Klimuk et al., 1999; Antohe et al., 2004; Hua, 2013). Importantly, all types of liposomal delivery systems are subjected to the EPR effect, with PEGylated liposomes having an advantage due to having reduced RES clearance and extended circulation time (Sawant and Torchilin, 2012).

### The accelerated blood clearance (ABC) phenomenon

The interaction of liposome components with the immune system has contributed to the challenges in translation to clinical use. Synthetic modifications to enhance their utility as drug delivery vehicles can result in antibody production against their various components and/or the encapsulated cargo. For example, repeated injection of PEGylated liposomes has been associated with loss of their long circulating properties and subsequent clearance from the blood (Dams et al., 2000; Ishida et al., 2003, 2006b). This phenomenon is known as the “accelerated blood clearance” (ABC) phenomenon. The ABC phenomenon is a major concern for the clinical application of PEGylated formulations that require multiple dosing regimens. Dams et al. first observed the ABC phenomenon by demonstrating that prior dosing of empty PEGylated liposomes influences the pharmacokinetics and biodistribution of the second dose of liposomes in rats and rhesus monkeys, when the doses were administered with an interval of 7 days (Dams et al., 2000). As a result, the circulation time of the second dose of PEGylated liposomes was significantly reduced, and liposome accumulation in the liver and spleen increased (Dams et al., 2000). Subsequent investigations have verified these findings, with a maximum clearance of liposomes 4–7 days after the initial dose in rats and 10 days in mice (Ishida et al., 2003, 2006b).

The exact mechanism underlying the ABC phenomenon is unclear. This phenomenon is affected by lipid dose, PEG surface density, and the interval between the first and consecutive injections (Ishida and Kiwada, 2008). Repeated injection of empty PEGylated liposomes in rats has been shown to elicit marked anti-PEG IgM production (Ishida et al., 2006b). This immune response is thought to be mediated by the spleen, as the degree of anti-PEG IgM production and the ABC phenomenon is dramatically decreased in splenectomized rats (Ishida et al., 2006a). Interestingly, administration of higher doses of the initial PEGylated liposomes (>1 μmol phospholipids/kg) have been shown to reduce the magnitude of the ABC phenomenon (Ishida et al., 2005). Increasing the phospholipid dose has been suggested to cause PEG-reactive B cells to become apoptotic, reducing anti-PEG IgM production and thus abating the ABC phenomenon (Ishida et al., 2006b; Ishida and Kiwada, 2008). The ABC phenomenon has not been reported to occur in patients receiving PLD, even after multiple-dosing regimens (Laverman et al., 2001). Generally higher doses (15 μmol phospholipid/kg) are administered clinically, which may account for this absence of the ABC phenomenon (Lyass et al., 2000). In addition, this response may also be due to doxorubicin-mediated macrophage death and the inhibition of B-cell proliferation and/or the death of proliferated B-cells (Ishida et al., 2006b; Szebeni and Moghimi, 2009).

### Complement activation–related pseudoallergy (CARPA)

Some liposomal systems are able to trigger the innate immune response, with subsequent activation of the complement system to trigger an acute hypersensitivity syndrome known as complement activation–related pseudoallergy (CARPA). The complement system is part of the innate immune response, and is involved in a range of immunological and inflammatory processes (Moghimi and Hunter, 2001). A relatively high percentage of patients (2–45%) have been reported to develop infusion-related hypersensitivity reactions to liposomal drug therapy. In addition, CARPA has been reported with both experimental and clinically approved liposomal formulations (e.g., Doxil®, Ambisome, and DaunoXome®) (Szebeni, 2005; Szebeni and Moghimi, 2009). CARPA is an immediate, non-IgE-mediated hypersensitivity reaction that involves symptoms such as anaphylaxis, facial flushing, facial swelling, headache, chills, and cardiopulmonary distress (Szebeni, 2005—the latter of which may limit the clinical use of potentially reactogenic liposomes in cardiac patients (Szebeni and Barenholz, 2009). General clinical management involves slowing the infusion rate or ceasing therapy, as well as the use of standard allergy medications (e.g., antihistamines, epinephrine, and corticosteroids).

This pseudoallergy is thought to be partly due to the activation of the complement system with the subsequent generation of C3 split-products (e.g., C3d) (Dempsey et al., 1996) and anaphylatoxins C3a and C5a (Szebeni, 2005; Szebeni and Moghimi, 2009). Binding of anaphylatoxins to their specific receptors on immune cells (e.g., mast cells, basophils, and macrophages) elicits the release of a multitude of vasoactive mediators, including histamine, tryptase, platelet-activating factor (PAF), leukotrienes (e.g., LTB_2_, LTB_4_, LTC_4_, LTD_4_, LTE_4_), thromboxane A2 (TXA_2_), and prostaglandins (e.g., PGD_2_). Initial activation triggers include the binding of IgG, IgM, C reactive protein (CRP), C1q, C3, and potentially, mannose binding lectin (MBL) and ficolin, to the liposomal vesicles (Szebeni and Barenholz, 2009). It should be noted that the sensitivity of different species to liposomal CARPA shows substantial variation, with some species (dogs and pigs) also showing tachyphylaxis (tolerance induction) following additional doses (Szebeni et al., 1999, 2007). Therefore, desensitization protocols using empty liposomes may be used to prevent CARPA, as well as pre-administration of complement inhibitors (e.g., soluble C receptor type 1, anti-C5 antibody, and indomethacin) (Szebeni and Barenholz, 2009).

All types of liposomes can activate the complement system. Liposome size, morphology, charge, lipid composition, bilayer packaging, surface characteristics, and administered lipid dose all regulate complement activation (Szebeni and Moghimi, 2009). Specific liposomal characteristics that enhance the propensity for complement activation include a positive or negative surface charge, increasing size, lack of liposomal homogeneity, endotoxin contamination, presence of aggregates, presence of drugs that can bind to and aggregate liposomes/lipids, presence of cholesterol in the bilayer membrane at ≥70%, and PEGylation with PEG-PE (Szebeni and Barenholz, 2009). Based on these findings, neutral small unilamellar vesicles have been shown to be the least reactogenic of the liposomal platforms (Szebeni and Barenholz, 2009). Formulation strategies to minimize the immunogenicity of liposomes have included methylation of the anionic charge localized on the phosphate oxygen of mPEG-phospholipid conjugate (Moghimi et al., 2006) or the use of other non-ionic lipopolymers and lipid conjugates, such as mPEG-substituted synthetic ceramides (Webb et al., 1998; Szebeni and Moghimi, 2009). The development of immunogenic reactions to liposomal therapies may lead to altered pharmacokinetics, loss of efficacy, and the rise of potentially serious toxicities (e.g., anaphylaxis) (Szebeni and Moghimi, 2009). This should therefore be considered in formulation design and closely monitored for in clinical practice.

## Experimental use of liposomes for biomedical applications

The application of liposomes in medicine offers significant prospects for novel and effective treatments in a wide range of pathological conditions. Since the discovery of liposomes over 50 years ago, there has been a significant increase in lipid–based drug delivery research at the experimental *in vitro* and *in vivo* phase. Liposomes have been utilized as a drug delivery carrier for a wide range of therapeutic compounds and diagnostic agents, such as drug molecules, gene therapy and bioactive agents (Hua and Wu, 2013). Modifications of these formulations are constantly being investigated in an effort to improve efficacy, reduce RES clearance and minimize toxicity—this includes changes in lipid composition, charge, and the addition of surface coatings and ligands (Hua and Wu, 2013; Monteiro et al., 2014). More recent strategies to improve on conventional or stealth liposomal systems involve active targeting, charged lipids, triggered release, and multi-functional formulations (Puri et al., 2009; Allen and Cullis, 2013; Bozzuto and Molinari, 2015).

Active targeting approaches with the conjugation of targeting ligands to the surface of liposomes have been extensively studied at the experimental level for a variety of biomedical applications, and particularly following parenteral administration (e.g., intravenous and intraperitoneal injection) (Torchilin, 1994; Vingerhoeds et al., 1994; Willis and Forssen, 1998; Noble et al., 2004; Deshpande et al., 2013; Hua and Cabot, 2013; Rip et al., 2014; Hua et al., 2015). Targeting ligands are used to increase the specificity of delivery of encapsulated cargo to and retain it in diseased tissues and cells, with minimal deposition in non-target sites. The notion that ligand-targeted liposomes have a therapeutic advantage over non-targeted liposomes is still subject to debate, with conflicting results in the literature (Ferrari, 2005; Puri et al., 2009; Riehemann et al., 2009). A number of studies have demonstrated enhanced uptake and efficacy of ligand-targeted liposomes in diseased tissue in comparison to non-targeted liposomes *in vivo* (Vingerhoeds et al., 1994; Puri et al., 2009; Allen and Cullis, 2013; Kraft et al., 2014). For example, attachment of folate to liposomes showed enhanced biodistribution of liposomes in folate-expressing tumors in a murine model (Gabizon et al., 2003). In addition, attachment of ICAM-1 monoclonal antibodies to the surface of loperamide-encapsulated liposomes, demonstrated increased efficacy and localization of the targeted nanoparticles to peripheral inflammatory tissue in a rodent model of musculoskeletal pain (Hua and Cabot, 2013). Conversely, there are studies that have shown no difference in the biodistribution and target tissue accumulation of ligand-targeted liposomes compared with non-targeted liposomes. For example, conjugation of HER2 monoclonal antibody fragments to liposomes did not increase the tumor localization of the nanoparticles, with both targeted and non-targeted liposomes achieving similarly high levels of tumor tissue accumulation (7–8% injected dose/g tumor tissue) in HER2-overexpressing breast cancer xenografts models (Kirpotin et al., 1997, 2006). However, doxorubicin-loaded anti-HER2 immunoliposomes produced significantly superior therapeutic results in comparison to all other control groups, including free doxorubicin, non-targeted liposomal doxorubicin and recombinant anti-HER2 Mab trastuzumab (Park et al., 2002). The mechanism of this enhanced anti-tumor efficacy was clearly not due to enhanced accumulation via antigen binding, but rather the result of the marked difference in pharmacodynamics of the targeted liposomal formulation *in vivo*, by mediating intracellular drug delivery to HER2-overexpressing cancer cells (Kirpotin et al., 2006). Therefore, it is likely that attachment of targeting moieties enhances therapeutic efficacy by increasing receptor-mediated uptake of drug-encapsulated liposomes into target cells, subsequent to the accumulation of the nanocarriers in the diseased tissues (Kirpotin et al., 2006; Puri et al., 2009).

Despite the improved biodistribution and therapeutic outcomes of ligand-targeted liposomes in a number of preclinical studies, the advantages have so far been negligible in the clinical research phase. Possible reasons for this discrepancy have previously been reviewed (Sawant and Torchilin, 2012; Allen and Cullis, 2013), and include factors such as disease-dependent anatomical and physiological barriers, target accessibility and expression, and formulation stability. The optimal targeting ligand density on the surface of each liposome still remains to be resolved, and will likely depend on characteristics of the molecular target (e.g., location, expression, internalization rate, and immunogenicity) (Puri et al., 2009; Hua and Wu, 2013; Kraft et al., 2014). In addition, detailed analysis of the degree of liposome accumulation, cellular internalization, intracellular functionality and intracellular degradation will be important parameters for clinical validation and translation (Puri et al., 2009). Through extensive experimentation, we are gaining a better understanding of the more appropriate clinical indications for ligand-targeted liposomal formulations.

Furthermore, modifications of the lipid bilayer with charged lipids have also attracted much attention (Bozzuto and Molinari, 2015). Addition of charged lipids to the liposomal bilayer has played an important role in developing bioadhesive, mucoadhesive and nucleic acid-based delivery systems. For example, modifying the surface charge of nano-delivery systems can influence the electrostatic interaction of the nanocarriers with components in the gastrointestinal (GI) tract following oral administration, and theoretically should confer selectivity to diseased tissue (Hua et al., 2015). Cationic nano-delivery systems have been shown to adhere to the mucosal surface within inflamed GI tissue, due to the interaction between the positively charged nanocarrier and the negatively charged intestinal mucosa (Coco et al., 2013). Colonic mucins carry a negative charge since their carbohydrates are substituted with numerous sulfate and sialic acid residues (Larsson et al., 2009; Antoni et al., 2014). Conversely, anionic nano-delivery systems preferentially adhere to inflamed GI tissue via electrostatic interaction with the higher concentration of positively charged proteins in inflamed regions. In particular, high amounts of eosinophil cationic protein and transferrin have been observed in inflamed colon sections in patients with inflammatory bowel disease (IBD) (Carlson et al., 1999; Peterson et al., 2002; Tirosh et al., 2009). Cationic nano-delivery systems are also able to effectively transport large, charged structures, such as DNA and RNA, based on electrostatic interaction between the positively charged phospholipids [e.g., dioleoylphosphatidylethanolamine (DOPE)] and negatively charged nucleic acids (Felgner et al., 1987; Campbell et al., 2002, 2009; Kunstfeld et al., 2003; Wu et al., 2007). Such liposomes have also been demonstrated to have greater interaction with tumor vessels due to the overexpression of negatively charged functional groups on the angiogenic endothelial cell membrane (Ran et al., 2002). It should be noted however, that there is a potential for electrostatic interactions and subsequent binding of these charged nanoparticles with other charge-modifying substances in the circulation or during GI transit (Hua et al., 2015). In addition, recent studies have identified potentially toxic *in vitro* and *in vivo* effects with the use of cationic lipids and polymers, including cell shrinking, reduced number of mitoses, vacuolization of the cytoplasm, and detrimental effects on key cellular proteins (e.g., protein kinase C) (Lv et al., 2006). For cationic lipids, the cytotoxic effects are determined by the structure of its hydrophilic group, with quaternary ammonium amphiphiles being more toxic than their tertiary amine counterparts. Inclusion of a heterocyclic ring has been shown to spread the positive charge of the head-group, thus attenuating the toxicity level (Lv et al., 2006).

Another approach to improve therapeutic efficacy of liposomal formulations has been to use triggering modalities for site-specific release of therapeutics from liposomes (Bibi et al., 2012). Strategies that have been utilized include remote triggers (e.g., temperature, ultrasound, magnetic, and light) and local triggers specific to the target site (e.g., enzymes and pH), through the use of specific lipid compositions and coatings (Guo and Szoka, 2003; Andresen et al., 2004; Ponce et al., 2006; Bibi et al., 2012). Of these strategies, the use of an external hyperthermic trigger to release therapeutic compounds from liposomal formulations (e.g., ThermoDox®) appears to be the most promising to date (Needham et al., 2000). Thermosensitive liposomes are modified with temperature-sensitive lipids (e.g., 1,2-distearoyl-sn-glycero-3-phosphocholine, DSPC) and/or polymers [e.g., poly(N-isopropylacrylamide)], which enables the nanocarrier to remain stable and retain their contents at physiologic temperatures. Upon heating, these liposomes undergo a phase change that makes them more permeable, causing the release of their cargo (Kono, 2001). Recent studies have investigated the use of cationic thermosensitive liposomes (CTSL) for tumor targeting (Dicheva et al., 2013, 2014), with promising results showing doxorubicin-encapsulated CTSLs having three-fold higher accumulation at the target site compared to the thermosensitive liposomal formulation (Dicheva et al., 2014). Translation of these drug delivery systems into the clinic has not yet been successful, with issues surrounding therapeutic efficacy (e.g., location of diseased tissue and accessibility for remote triggers) and potential toxicity of particularly synthetic components of the drug delivery system (Bibi et al., 2012; Allen and Cullis, 2013). Overall, this technology is promising and does warrant further investigation to determine which disease states would benefit from this localized treatment platform and how to make such formulations safer for clinical use.

Finally, a number of experimental studies have focussed on complex multi-functional liposomal formulations in an attempt to develop more efficient drug delivery systems. These include liposomal formulations that combine one or more of the following strategies—active targeting with one or more targeting ligands, response to triggers to control drug release, delivery of a combination of therapeutics (e.g., siRNA and small molecule drugs), and biomarker and imaging capabilities (Zhang et al., 2011; Allen and Cullis, 2013; Charron et al., 2015; Cole and Holland, 2015). In particular, theranostic nanoparticles have generated much interest as it is both a therapeutic and diagnostic tool all-in-one (Figure 1). A typical theranostic delivery system would include the nanoparticle, imaging component, targeting ligand, and therapeutic agent. A number of studies have shown effective diagnostic imaging and therapeutic delivery of encapsulated drugs *in vivo* with theranostic nanosystems, especially in various cancers (Charron et al., 2015; Cole and Holland, 2015). For example, Han et al. (2014) conjugated ECl-GLuc to nickel-chelating liposomes (ECl-GLuc-liposome), and demonstrated significant bioluminescence imaging and targeted drug delivery in both SKOv3 cells *in vitro* and in ErbB2-overexpressing metastatic ovarian tumors *in vivo* in a murine model (Han et al., 2014). ECl-GLuc is a recombinant protein generated by fusing the ECl peptide (an artificial ligand of ErbB2) with Gaussia luciferase (GLuc). Although innovative and efficient, translation of multi-functional drug delivery systems to the clinic would need to show significant therapeutic advantage over other therapeutic strategies, due to the added costs and complexities required in the manufacturing process. In addition, multi-functional systems will need to address the potential mismatch between the doses required for the effective use of each component in patients, for example imaging and therapy for theranostic nanosystems (Teli et al., 2010). At this stage, packaging multiple payloads in the same carrier would appear most promising, however sequencing and scale up of this kind of approach would still be challenging (Zhang et al., 2011; Allen and Cullis, 2013).

## Clinically approved liposomal-based therapeutics

Many liposomal products are on the market with more in clinical development. Some of the most successful delivery methods rely on PEG conjugated lipids. In fact, the first FDA approved nano-drug, doxorubicin, is delivered using PEGylated liposomes (Ning et al., 2007). Often used in combination with other medicines, PLD treats various types of cancer including AIDS-related Kaposi's sarcoma, leukemia, and ovarian, breast, bone, lung, and brain cancers. PLD has also been found to be an effective alternative to conventional doxorubicin in patients with pre-existing cardiac dysfunction (Schmitt et al., 2012). When doxorubicin is incorporated in PEGylated liposomes, it minimizes the uptake and clearance by the RES, which prolongs the serum and plasma half-life. This allows the PLD to accumulate in the tumor tissue, rather than in non-target healthy tissues (Rahman et al., 2007). Furthermore, the use of PLD ensures that doxorubicin can pass through the myocardium without being released and contributing to cardiac muscle cell toxicity (Rahman et al., 2007). Finally, PLD avoids the high plasma peak levels of free drug, which has been correlated with cardiotoxicity (Lyass et al., 2000). In addition to receiving FDA approval for usage of PLD in 1995, combination therapies of PLD and other drugs (such as bortezomib for the treatment of relapsed or refractory multiple myeloma) have recently received FDA approval (Ning et al., 2007). Another type of PEGylated liposome currently in Phase I trials is PEPO2, an irinotecan-encapsulated liposomal formulation used to treat advanced refractory solid tumors (Chang et al., 2015). Camptothecin, which is formulated in PEGylated stealth liposomes, is also in Phase I trials for ovarian cancer treatment (Zamboni et al., 2009).

In addition to PEG conjugated lipids, conventional and cationic liposomal-based drugs have also been FDA approved. Liposomal amphotericin B for anti-fungal prophylaxis (Chandrasekar, 2008), daunorubicin for the treatment of leukemia and solid tumors (Chang and Yeh, 2012), verteporfin to treat macular degeneration (Chang and Yeh, 2012), cytarabine or cytosine arabinoside to treat neoplastic meningitis and lymphomatous meningitis (Chang and Yeh, 2012; Jahn et al., 2015), and morphine sulfate for pain management are currently on the market (Chang and Yeh, 2012). Regardless, many of these drugs are still undergoing clinical trials to test their effects of dose escalation and therapeutic efficacy (Chang and Yeh, 2012). For example, liposomal amphotericin B is in a prospective Phase II trial to test the safety and tolerability of high doses (Giannella et al., 2015). Advantages of these marketed drugs include a reduced toxicity by increased vasculature permeability/accumulation at the target tissue and an ability to encapsulate drugs of different lipophilicities while protecting them from biodegradation (Immordino et al., 2006; Chang and Yeh, 2012; Allen and Cullis, 2013).

Many more liposomal-based drugs are in various stages of clinical development to test their pharmacokinetics and biodistribution profiles. These include irinotecan SN-38 in Phase I/II to treat colorectal cancer (Zhang et al., 2004; Suenaga et al., 2015) and a liposomal-based all-trans-retinoic acid (ATRA) in Phase II that contains the drug tretinoin to treat acute promyelocytic leukemia and hormone-refractory prostate cancer (Ozpolat et al., 2003). One type of conventional liposomal formation is EndoTAG-1, which carries paclitaxel. It is embedded in a cationic liposome and is in Phase II trials to treat advanced triple-negative breast cancer (Awada et al., 2014) and pancreatic cancer (Löhr et al., 2012). EndoTAG-1 is prepared in a 50:47:3 molar ratio of 1,2-dioleoyl-3-trimethylammonium propane (DOTAP), 1,2-dioleoyl-sn-glycero-3-phosphocholine (DOPC), and paclitaxel (Chang and Yeh, 2012). EndoTAG-1 exhibits a greater antivascular effect on tumor vasculature while the usage of DOTAP, a cationic synthetic lipid, in EndoTAG-1 allows for selective affinity to the target tumor (Chang and Yeh, 2012). Another form of paclitaxel, LEP-ETU (liposome-entrapped paclitaxel easy-to-use), is in Phase I/II trials (Zhang et al., 2005; Immordino et al., 2006). LEP-ETU is prepared in a 90:5:5 molar ratio of DOPC, cholesterol, and cardiolipin (Chang and Yeh, 2012). Higher doses of LEP-ET can be safely administered compared to paclitaxel alone (Fetterly et al., 2008). Additionally, the use of cholesterol and cardiolipin allows for greater stability and reduced cardiotoxicity, respectively (Chang and Yeh, 2012). DOPC is a recently developed neutral liposome carrier for siRNA delivery and is currently in clinical testing (Mangala et al., 2009). Another drug in Phase I/II trials is annamycin to treat acute lymphocytic leukemia (Wetzler et al., 2013). With non-cross resistance properties and an enhanced cellular uptake and retention, annamycin's efficacy and anti-tumor activity is enhanced with the use of a targeted liposomal-based delivery system (Zou et al., 1994). These and more liposomal-based drugs that are FDA approved or currently in clinical trials are summarized in Table 1.

**Table 1:** **Marketed liposomal-based therapeutics and products in clinical development**.

**Drug**	**Disease**	**Status**	**Type of liposomal-based delivery system**	**Source(s)**
Paclitaxel LEP-ETU	Advanced triple-negative breast cancer	Phase I/II	siRNA	Zhang et al., 2005; Immordino et al., 2006
siRNA	Ovarian cancer	Phase I	DOPC neutral liposomes	Mangala et al., 2009
Paclitaxel EndoTAG-1	Advanced triple-negative breast cancer	Phase II	Cationic	Chang and Yeh, 2012; Awada et al., 2014
Paclitaxel EndoTAG-1	Pancreatic cancer	Phase II	Cationic	Löhr et al., 2012
Mitoxantrone LEM-ETU	Acute myeloid leukemia, multiple sclerosis, and prostate cancer	Phase I	Cationic	Immordino et al., 2006; Chang and Yeh, 2012
Verteporfin	Molecular degeneration	FDA Approved in 2000	Cationic	Chang and Yeh, 2012; Allen and Cullis, 2013; Gross et al., 2013
Amikacin	Lung infection	Phase II/III	Conventional	Chang and Yeh, 2012; Clancy et al., 2013; Olivier et al., 2014
Vincristine	Non-Hodgkin lymphoma	FDA Approved in 2012	Conventional	Allen and Cullis, 2013; Wang et al., 2015
Tretinoin	Acute promyelocytic leukemia and hormone-refractory prostate cancer	Phase II	Conventional	Ozpolat et al., 2003; Immordino et al., 2006
Irinotecan SN-38	Metastatic colorectal cancer	Phase I/II	Conventional	Zhang et al., 2004; Suenaga et al., 2015
Annamycin	Acute lymphoblastic leukemia	Phase I/II	Conventional	Wetzler et al., 2013
Amphotericin B	Anti-fungal prophylaxis	FDA approved in 1997	Conventional	Chandrasekar, 2008; Allen and Cullis, 2013
Daunorubicin	Leukemia and solid tumors	FDA Approved in 1996	Conventional	Chang and Yeh, 2012; Allen and Cullis, 2013
Cytarabine or cytosine arabinoside	Neoplastic meningitis and lymphomatous meningitis	FDA Approved	Conventional	Chang and Yeh, 2012; Jahn et al., 2015
Morphine sulfate	Pain Management	FDA Approved in 2004	Conventional	Chang and Yeh, 2012; Allen and Cullis, 2013
Lurtotecan	Ovarian cancer, head, and neck cancer	Phase I/II	Conventional	Dark et al., 2005; Chang and Yeh, 2012
Vinorelbine	Newly diagnosed or relapsed solid tumors	Phase I	Conventional	Allen and Cullis, 2013
Topotecan	Advanced solid tumors	Phase 1/II	Conventional	Seiden et al., 2004; Allen and Cullis, 2013
Nystatin	Fungal Infections	Phase I/II	Conventional	Offner et al., 2004
Doxorubicin	Leukemia, breast cancer, bone cancer, lung cancer, brain cancer	FDA Approved in 1995	PEGylated	Ning et al., 2007
Doxorubicin and bortezomib	Relapsed or refractory multiple myeloma	FDA Approved in 2007	PEGylated	Ning et al., 2007
Thermosensitive doxorubicin	Liver tumors	Phase III	PEGylated	Yarmolenko et al., 2010
Thermosensitive doxorubicin	Chest wall recurrences of breast cancer	Phase I	PEGylated	Yarmolenko et al., 2010
Irinotecan	Advanced refractory solid tumors and colorectal cancer	Phase I	PEGylated	Chang et al., 2015
Camptothecin analog	Ovarian cancer	Phase I	PEGylated	Zamboni et al., 2009

## Why the bottleneck for translation into clinical practice?

Despite considerable research in the last 50 years, the clinical translation of liposome assisted drug delivery platforms has not progressed as quickly as the plethora of positive results would have suggested. Liposomal formulations have demonstrated significant therapeutic advantages for a multitude of biomedical applications, however the major reasons for the bottleneck have been attributed to issues surrounding pharmaceutical manufacturing, government regulations and intellectual property (IP). Similar obstacles are faced by other nano-delivery systems for translation into the clinic (Allen and Cullis, 2004, 2013; Zhang et al., 2008; Sawant and Torchilin, 2012; Narang et al., 2013). Limitations in pharmaceutical development are centered on quality assurance and cost. Quality assurance involves issues surrounding the manufacturing process and stability of the formulation, with nano-delivery systems being affected by (i) scalability of the manufacturing process, (ii) reliability and reproducibility of the final product, (iii) lack of equipment and/or in-house expertise, (iv) chemical instability or denaturation of the encapsulated compound in the manufacturing process, and (v) long term stability issues (Narang et al., 2013). Suitable methods for the industrial scale production of conventional liposomes have been successfully developed, without the need for numerous manufacturing steps or the use of organic solvents (Jaafar-Maalej et al., 2012; Kraft et al., 2014). The challenges arise when the functionality of the liposomal delivery system becomes more complex, such as the addition of surface modification with coatings and/or ligands. Integration of multiple components to a single nanosized carrier requires multiple chemical synthesis steps and formulation processes, which inevitably pose problems for large scale good manufacturing (cGMP) production, increases the cost of production, and makes the evaluation of such products more difficult (Teli et al., 2010; Tinkle et al., 2014). Increasing the number of physicochemical variables in a nanoformulation system also makes it more complicated to assess the pharmacokinetics, pharmacodynamics and toxicology of a formulation following administration (Teli et al., 2010; Tinkle et al., 2014). For example, the use of synthetic coatings and ligands may affect the biocompatibility, biodistribution and toxicology profile of liposomal formulations, and will require further evaluation to understand the interaction of the nanoparticles with biological tissues and cells (Allen and Cullis, 2004, 2013; Zhang et al., 2008; Sawant and Torchilin, 2012; Narang et al., 2013; Tinkle et al., 2014). Improvements in the regulatory framework for the assessment of nanoformulations will require consultation with academia and industry.

IP of liposomal based therapies can be a perplexing issue and is likely to contribute to increasing development costs. IP strategies may vary depending on various factors including non-targeted or targeted liposomal formulations, the design and composition of the liposomes, and drugs that may be encapsulated. Any of these factors may contribute to a weak IP position and reduce the commercial attractiveness of the formulation—this will have implications on further development of the product in the research and development pipeline. Given the complexities of incorporating nanotechnology into biomedical applications, there will likely be multiple patents associated with any given technology and the need for cross-licensing arrangements (Murday et al., 2009). It will be important to simplify the pathway from invention to commercialization through new IP practices and protocols, so as to reduce the time and expense required for negotiating collaboration and licensing agreements (Murday et al., 2009).

Finally, clinical trials of liposomal formulations are generally more complex than conventional formulations, as a number of control groups are required to account for different aspects of the drug delivery system. In order to get to this stage, the formulation has to first pass pharmaceutical and commercial qualities as discussed above. Then we can determine whether therapeutic efficacy in preclinical animal studies translate to success in humans (Allen and Cullis, 2004, 2013; Narang et al., 2013). Even at this stage, the cost-benefit analysis may be a limitation to the clinical translation of some liposomal based therapies when compared to an approved counterpart or existing therapies, in terms of efficacy or drug-related side effects.

## Conclusion

The application of liposomes to assist drug delivery has already had a major impact on many biomedical areas. Understanding the advances in liposomal technology to date and the challenges that still need to be overcome, will allow future research to improve on existing platforms and to address the current translational and regulatory limitations. Continued translational success will require communication and collaboration between experts involved in all stages of pharmaceutical development of liposomal technologies, including manufacturing and pharmaceutical design, cellular interactions and toxicology, as well as preclinical and clinical evaluation.

### Conflict of interest statement

The authors declare that the research was conducted in the absence of any commercial or financial relationships that could be construed as a potential conflict of interest.
